# Insights Into the Biogenesis and Emerging Functions of Lipid Droplets From Unbiased Molecular Profiling Approaches

**DOI:** 10.3389/fcell.2022.901321

**Published:** 2022-06-08

**Authors:** Miguel Sánchez-Álvarez, Miguel Ángel del Pozo, Marta Bosch, Albert Pol

**Affiliations:** ^1^ Cell and Developmental Biology Area, Centro Nacional de Investigaciones Cardiovasculares (CNIC), Madrid, Spain; ^2^ Lipid Trafficking and Disease Group, Institut d'Investigacions Biomèdiques August Pi i Sunyer (IDIBAPS), Barcelona, Spain; ^3^ Department of Biomedical Sciences, Faculty of Medicine, Universitat de Barcelona, Barcelona, Spain; ^4^ Institució Catalana de Recerca i Estudis Avançats (ICREA), Barcelona, Spain

**Keywords:** lipid droplets, high-content screening, functional genomics, lipidomics, proteomics, proteostasis, innate immunity

## Abstract

Lipid droplets (LDs) are spherical, single sheet phospholipid-bound organelles that store neutral lipids in all eukaryotes and some prokaryotes. Initially conceived as relatively inert depots for energy and lipid precursors, these highly dynamic structures play active roles in homeostatic functions beyond metabolism, such as proteostasis and protein turnover, innate immunity and defense. A major share of the knowledge behind this paradigm shift has been enabled by the use of systematic molecular profiling approaches, capable of revealing and describing these non-intuitive systems-level relationships. Here, we discuss these advances and some of the challenges they entail, and highlight standing questions in the field.

## Introduction

Lipids constitute essential building blocks for cell membrane structures, powerful sources of energy through β-oxidation, and modulators of cell compartment transactions and cell signaling and behavior (Alberts et al., 2014). However, several lipid species can be highly toxic for cells when in excess and/or in free form: the aberrant accumulation of cholesterol and free fatty acids disturbs the functioning of organelles such as mitochondria, lysosomes or the endoplasmic reticulum (ER), and can trigger apoptotic cascades when stress is sustained (Trajkovic et al., 2008; Bosch et al., 2011; Tam et al., 2018; Enrich et al., 2019; Höglinger et al., 2019). They are also immediate targets for the propagation of oxidative damage upon accumulation of ROS through peroxidation (Dierge et al., 2021).

Lipid droplets—LDs, also labelled *lipid bodies;* or *adiposomes* in early studies—constitute a safe and efficient means to store lipids, particularly for organisms exposed to environments with intermittent nutrient availability. They appeared early in evolution and can be found across all eukaryotic phyla studied, and in virtually all cell types of higher metazoans (Lundquist et al., 2020); LD-like organelles have also been described for a number of prokaryotes, including *Mycobacteria* species (Daniel et al., 2004; Wältermann et al., 2005; Lundquist et al., 2020). LDs consist of accumulations of neutral lipids (a predominant share, triacylglycerides (TGs) and cholesterol esters) surrounded by a single leaflet of phospholipids with their hydrophobic tails facing the neutral lipid core, and their hydrophilic heads contacting the aqueous environment of the cytosol (Fujimoto and Parton, 2011; Olzmann and Carvalho, 2019). LD biogenesis is still only partially understood; three different mechanistic models have been proposed to date. In the prevailing “budding model”, cytoplasmic LDs are generated from TG and sterol ester accumulations between the two membrane leaflets of the ER that phase-separate onto a neutral lipid “lens” (Thiam and Ikonen, 2021), which buds towards the cytoplasmic leaflet (which eventually will constitute the phospholipid coat of the LD) in a process tightly regulated by specific proteins such as seipin (Wang et al., 2016; Chorlay et al., 2019; Santinho et al., 2020; Prasanna et al., 2021). This process might take place preferentially at specific ER subdomains with a certain curvature such as ER tubules (Santinho et al., 2020). Two alternative models were developed, largely to accommodate independent ultrastructural studies. The “egg-cup” model proposes that LDs (the egg) develop at specialized ER “depressed” regions (the cup), where adipophilin clusters would enable the transfer of lipids from the ER to the LD (Robenek et al., 2006). The “enfolding” model hypothesizes that loops of ER membrane successively incorporate onto LDs, explaining the observation of ER-like membranes and associated ribosomal particles within LDs of monocytes; this model might require further evidence from 3D reconstructions (Wan et al., 2007). These different models might be envisioned as compatible, depicting distinct LD biogenesis stages.

Different protein subsets, including enzymes governing LD growth and usage (such as acyl glycerol transferases and lipases, respectively) are localized to the crowded surface of the LD, through mechanisms still undergoing characterization (Olzmann and Carvalho, 2019; Song et al., 20212021; Olarte et al., 2022). Recent studies describe similar LD structures forming either from the outer nuclear membrane of the nuclear envelope (presumably as a protective mechanism against an imbalance in lipid composition and relative fatty acid saturation at the inner nuclear membrane) as well as from the inner nuclear membrane (Romanauska and Köhler, 2018; Romanauska and Köhler, 2021; McPhee et al., 2022); the biogenesis and function of these structures are relatively less characterized.

The particular ultrastructure of LDs, with a spherical core devoid of any molecules apart from triacylglycerides and cholesterol species, favored their long-standing view as relatively inert inclusions floating in the cytoplasm, passively subject to growth and consumption cycles (Napolitano and Fawcett, 1958; Fujimoto and Parton, 2011; Olzmann and Carvalho, 2019). This view has been modified in the postgenomic era. LDs are extremely dynamic structures, rapidly changing in number and size by orders of magnitude in response to different conditions, and engaging in physical and functional communication with different organelles apart from the ER, such as mitochondria, endosomal/lysosomal compartment or peroxisomes through specialized membrane contact sites (MCSs) (Herker et al., 2021; Rakotonirina-Ricquebourg et al., 2022). Evidence exists for different LD populations even within a same cell, with distinct functions (Eisenberg-Bord et al., 2018; Ugrankar et al., 2019). Further, unexpected prominent roles in proteostasis (Roberts and Olzmann, 2020) or host-pathogen interaction (Bosch et al., 2021a) have been identified. These conceptual breakthroughs of biomedical relevance have been in part enabled by technological advances allowing us to systematically interrogate the molecular composition (most notably, the LD proteome) and the genetic regulation of these organelles.

## LD Functional Genomics: Screening for LD Biogenesis, Function and Communication

### A “Photogenic” Organelle

**FIGURE 1:**
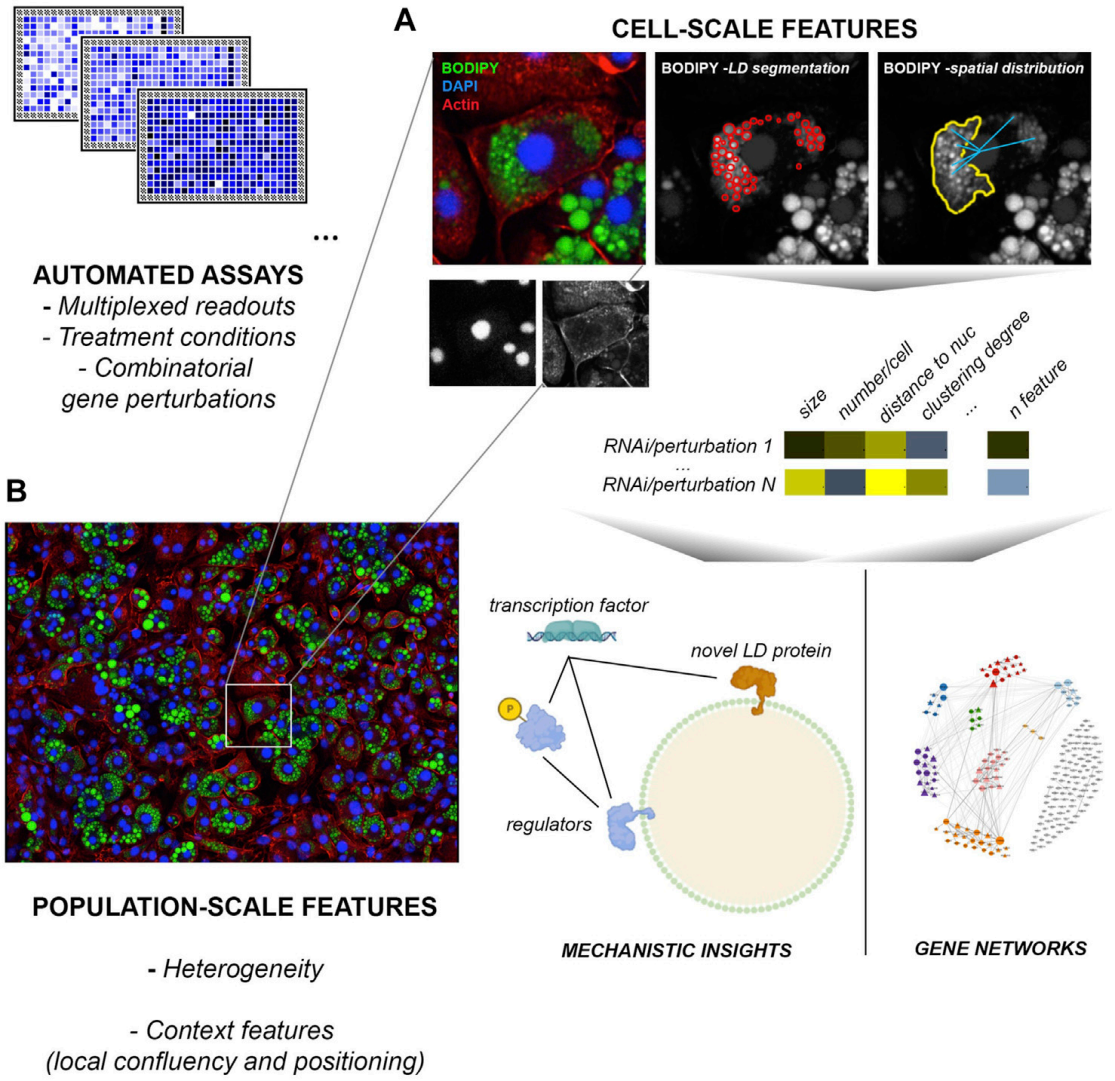
High-content screens applied to LD studies. Current platforms are highly multiplexable and allow for the automated acquisition and analysis of millions of images across genome-scale screens in a few days. **(A)** Cell-scale analysis of LD number, size, spatial distribution and relationship with other structures such as the nucleus or the actin cytoskeleton. Normalized features enable the identification of specific mechanisms required for LD formation and function in a given condition, and the inference of relationships between gene groups based on phenotypes to model gene regulatory networks. **(B)** Image-based readouts capture population and local context features (heterogeneity, local confluency etc) that can be highly informative to understand the role of LDs on tissue homeostasis and their regulation. The example image (Aboy et al., 2019) was acquired by MCM Aboy-Pardal (CNIC, Spain) from a culture of 3T3-L1 mesenchymal cells induced for adipose-like phenotypes, labeled with BODIPY FL 493/503 (green), actin cytoskeleton (red) and nuclei (blue).

While relatively labile under routine immunolabeling procedures involving cell permeabilization with detergents, LDs are cell structures particularly amenable for imaging and extraction of rich multiparametric information (number, size, subcellular distribution, relationship to other cell structures). Their characteristic spherical morphology and the availability of highly specific cell/tissue permeable dyes for neutral lipids (BODIPY probes, Nile Red stain, LIPIDTOX™) facilitate automated segmentation routines and the computing of their number, size and relative position across extensive datasets (Nioi et al., 2007; Gocze and Freeman, 1994; Bombrun et al., 2017; Greenspan et al., 1985; Zhao et al., 2022) (Figure 1). This kind of data can capture part of the complexity of the phenotypic state of cells and the impact of chemical or genetic perturbations therein, and can thus be very powerful to map gene networks and mechanisms involved in a given biological process (Liberali et al., 2014; Sailem and Bakal, 2017). Moreover, these studies can also capture cell- and population-context features, such as confluence, heterogeneity, spreading and morphology, or phenotypic signatures derived from other cell structures: information typically lost in other approaches (Liberali et al., 2015; Gut et al., 2018). Within-tissue heterogeneity in LD biogenesis, for example, is a striking but poorly understood phenomenon that could contribute to metabolic flexibility, and maximize protection from lipotoxicity (Herms et al., 2013). The very nature of these experimental platforms usually allows for interrogating in parallel the differential impact of a same collection of perturbations across distinct conditions or stimuli (Gut et al., 2018); for example, a given siRNA library targeting all known components of a metabolic pathway, or the whole kinome. Further, fluorescent probes designed from physiological substrates such as fatty acids (i.e. palmitate) or LDL-cholesterol, can provide additional information on the uptake, storage and release of these lipid species from LDs (Rambold et al., 2015; Scott et al., 2015; Vanharanta et al., 2020).

### Learning About LD Biology From Model Organisms and Genetics

Because LD formation and function are significantly conserved across eukaryotic phyla (Leyland et al., 2020; Lundquist et al., 2020), different systematic genetics applications on model organisms have been very useful to explore LD biology in physiologically relevant experimental contexts.

The baker’s yeast *Saccharomyces cerevisiae* is a first prominent example: the versatility and ease of use of this model unicellular eukaryote allows for the combinatorial interrogation of genetic interactions across the whole genome, using different readouts—from subcellular imaging to relative survival across different limiting growth conditions—; this allows for the precise mapping of genetic networks and enzyme complexes to as yet unparalleled resolution (Michel et al., 2017; Costa et al., 2018; Arita et al., 2021; Costanzo et al., 2021). The relative ease to systematically tag endogenous loci with fluorescent markers has also contributed to the relevance of these systems to organelle biology research (Meurer et al., 2018; Weill et al., 2018). It is difficult to overstate the contribution of yeast systematic genetics approaches to our current understanding of eukaryotic lipid metabolism and LD biology (Radulovic et al., 2013). A few recent examples include: insights onto the regulated dynamics and spatial positioning of LDs during nutrient stress conditions to supply free fatty acids (Henne et al., 2018; Hariri et al., 2019); the first mechanistic and structural descriptions of the seipin complex driving LD budding from the ER (Klug et al., 2021; Arlt et al., 2022), and systematic screens for its regulatory partners (Eisenberg-Bord et al., 2018); the description of specific mechanisms by which LDs contribute to manage protein aggregates (*see also below*) (Moldavski et al., 2015); or the discovery of a new class of LDs stemming from nuclear membranes (Romanauska and Köhler, 2018). Innovative applications of imaging technologies for automated screening, such as 3D imaging (Lv et al., 2019), have also been reported in this system.

*Caenorhabditis elegans* is a multicellular model system whose use in research largely developed during the birth of molecular biology (Nigon and Félix, 20172017). *C. elegans* has a very short life cycle, a translucent anatomy facilitating the direct visualization of subcellular structures across all life stages using different approaches including direct Nile red staining or label-free stimulated Raman scattering microscopy (Wang et al., 2011; Mak, 2013), and the possibility of performing RNAi screens *in vivo* at low cost directly from dsRNA-expressing bacterial libraries (Hammell and Hannon, 2012). One of the first genome-wide screen for regulators of lipid uptake, trafficking and accumulation in a multicellular organism was enabled by the ease of use of *C. elegans* (Ashrafi et al., 2003). In this analysis, 305 enhancers and 112 suppressors of body fat accumulation under standard growth conditions were identified, including several regulators of insulin-like signaling. Disruption of conserved neuroendocrine signaling, through depletion of serotonin biogenesis enzymes, a specific glutamate receptor, or the worm homolog of the obesity-related gene *Tubby* (Noben-Trauth et al., 1996; Ashrafi et al., 2003) altered fat accumulation, highlighting the relevance of non-cell autonomous mechanisms. Other metabolic pathways found in different studies as central to LD biogenesis such as phospholipid metabolism and fatty acid synthesis were also identified. Notably, the authors reported that disruption of β-oxidation decreases fat accumulation, a paradoxical observation that might be explained by a recent model whereby β-oxidation flux encodes information on fatty acid availability through ROS production to regulate lipolysis (Ding et al., 2021). Several hormone receptors controlling fatty acid and cholesterol metabolism, such as the HNF4a homolog, were also identified as hits. An independent small-scale RNAi screening study used stimulated Raman scattering microscopy as an alternative to the use of neutral lipid dyes, whose incorporation can present different degrees of variability (Wang et al., 2011). Upon interrogation of 272 cherry-picked genes encoding membrane signaling transducers and nuclear hormone receptors as proof of concept, together with known adiposity regulators such as *daf2*/IGF-1, the authors identified nine genes not previously linked to lipid accumulation, including *gcy-28*, a worm homolog of human natriuretic peptide receptor—which has indeed a role in humans in lipolysis control (Lafontan et al., 2008).

*C. elegans* has also been used to screen for small compounds regulating lipid accumulation across extensive libraries (Lemieux et al., 2011); this study subsequently combined GFP-gene reporter systems and genetic epistasis analysis across mutant strains for known pathways (fatty acid biosynthesis and insulin signaling) to explore the potential mechanisms by which the identified small compound hits exerted their effects. Notably, among ten selected strong hits altering lipid accumulation, only one displayed a clear genetic dependency on a specific regulator (the AMPK homolog *aak*) to exert its function. Recent *C. elegans* screening studies have also provided insights onto the regulation of the formation of nuclear LDs (nLD); apart from COPI components and the seipin homolog SEIP-1, the authors identified the nuclear inner membrane NEMP-1/Nemp1/TMEM194A as a required gene for nLD formation (Mosquera et al., 2021).

Despite exhibiting higher organismal complexity and homology for known human disease-related genes than the previous models, Drosophilidae have a significant caveat as compared to other model systems in the sense that arthropods are sterol auxotrophs, and lack most of the machinery for *de novo* biosynthesis of cholesterol from acetyl-CoA (Dobrosotskaya et al., 2002; Dobrosotskaya et al., 2003; Zhang et al., 2019), a process that bears relevance to understand certain aspects of LD dynamics in humans and other organisms (Ashrafi et al., 2003; Ta et al., 2012; Cadena Del Castillo et al., 2021). Nonetheless, the wealth of knowledge on fly genetics and the significant conservation of other aspects of LD biogenesis have made *Drosophila* systems useful for LD research. A first experimental context has been the use of larvae: beyond their relatively easy handling and the possibility to directly observe fluorescent markers, larvae have the peculiarity of being amenable for direct centrifugation; LDs accumulate due to their buoyancy on the upper part of the animal´s body cavity, making the assessment of relative LD content and the specific recruitment of proteins to LDs straightforward. This experimental setting has been used to systematically screen for regulators of lipid accumulation in *Drosophila* larvae, leading to the identification of Sir2—the fly SIRT1 homolog, a regulator of metabolic homeostasis and lifespan (Guarente, 2005)—as a key gene modulating the use of stored lipids to face nutrient starvation (Reis et al., 2010). An alternative strategy has relied on the screening of cORF overexpression libraries in larvae (Yao et al., 2018); this approach highlighted the chromatin remodeling complex MRT/PZG/NURF as important to regulate LD size through the transcriptional control of perilipin-1 levels.

Adult flies offer the advantage of presenting full development of a specialized organ, the fat body, which bears functional resemblance to liver and adipose tissues in humans and allows for detailed studies on LD subpopulation dynamics (Zheng et al., 2016; Ugrankar et al., 2019). A relevant finding from genome-scale inducible RNAi screens was the identification, among 77 significant hits, of store-operated calcium entry (SOCE, an ER-resident system that upon ER Ca^2+^ depletion, activates channels conducting extracellular Ca^2+^ towards the ER lumen) as an important regulator of lipid accumulation in adult flies (Baumbach et al., 2014). These observations harmonize with studies on obesity mouse models, which corroborate a tight relationship between ER homeostasis, calcium store management, and lipidostasis (Fu et al., 2011). The fact that seipin can localize to sites communicating ER and mitochondria to modulate mitochondrial Ca^2+^ import further supports the relevance of these relationships (Combot et al., 2022).

Recently, advanced tools for LD biology research have been developed for zebrafish as a vertebrate model organism (Lumaquin et al., 2021; Wilson et al., 2021). Live imaging and automation in this experimental system, which is amenable for systematic genome-scale or small compound library screening (Williams and Hong, 2016; Sun et al., 2020), have experienced a remarkable advance in recent years (Logan et al., 2018; Osmani and Goetz, 2019). These tools might contribute to our further understanding of the biological roles, especially regarding specific tissues and developmental stages, of LDs.

### Image-Based Functional Genomics of LD Formation and Function in Metazoan Cells

Among metazoan cell culture systems, *Drosophila* RNAi cell screens have historically provided a number of advantages: the fly genome has a ∼3-fold lower redundancy as compared to the human genome, facilitating the interpretation of results and minimizing paralog compensation effects, while exhibiting a remarkable conservation of key pathways and disease-related genes (Lloyd and Taylorflies, 2010). Further, genome-scale RNAi libraries can be quickly and inexpensively developed *in vitro* from genomic DNA amplicons without the need of enzymatic processing, and they can provide efficient and specific target knockdown in the absence of transfection reagents (Ramadan et al., 2007). A hallmark genome-scale RNAi screen was conducted on *Drosophila* hemocytes for regulators of LD formation, during loading with sodium oleate emulsions (Guo et al., 2008). The dimensionality of the analysis was deceivingly limited, as it included basically three features: LD number, size, and “dispersion”, meaning degree of homogeneous distribution in the cell as opposed to perinuclear clustering. However, it must be noted that the third, composite feature has the potential to capture extensive information on LD dynamics. Image analysis was mostly performed by two independent human observers by eye inspection; nonetheless, the study consistently identified novel pathways as involved for LD biogenesis, including phospholipid anabolism and Golgi-ER retrograde transport, in agreement with previous observations on worms (Ashrafi et al., 2003). Golgi-ER transport regulators are in fact required for specific protein subsets to reach the LD (Soni et al., 2009; Olarte et al., 2022). Remarkably, an independent contemporary study on *Drosophila* cells also identified COPI transport as an unexpected trafficking activity required for LD formation and homeostasis using image-based RNAi automated screens, further suggesting that altered lipolysis could contribute to the phenotype of few, large LDs observed when knocking down those gene subsets (Beller et al., 2008). Subsequent mechanistic studies confirmed these pathways as essential for LD formation in mammalian cells (Krahmer et al., 2011); other candidates have been followed up by other groups, unveiling unexpected relationships between pre-mRNA processing and LD dynamics (Bennick et al., 2019). Focused screenings across specific conditions have also found relationships of some of these pathways with other cell functions, such as cell cycle progression, phospholipid anabolism and ER homeostasis (Sanchez-Alvarez et al., 2014).

This system is also amenable for studying the relative distribution of proteins to LDs and its regulation. Using automated imaging for the subcellular distribution of GPAT4—an LD component that selectively targets mature LDs—, recent genome-wide screens identified a distinct ‘late’ ER-LD trafficking pathway (Song et al., 20212021), whereby ER exit sites (ERES) (Malhotra and Erlmann, 2015) are required to form a heterotypic communication between the ER and LDs, with the assistance of specific tether proteins such as the Syx5 and Bet1 SNAREs. The link of ERES with a specific mechanism for LD functional regulation is interesting, because of the role ERES themselves play in metabolism of lipids, especially cholesterol (Runz et al., 2006; Sun et al., 2007; Hayer et al., 2010). Emerging evidence of LD-associated proteins such as CIDE-B, regulating ERES function further suggests a complex reciprocal crosstalk between these cell compartments to modulate cell metabolism (Su et al., 2019).

Functional genomics on specific human cell models have also been developed and applied to gain systems-level insights onto organelle biology (Liberali et al., 2014; Liberali et al., 2015). Importantly, these approaches can provide information regarding *upstream* signaling pathways and cell functions influencing a given phenotype. Image-based genome-scale siRNA screens revealed that the Wnt3a ligand—a paralog of a family of secreted proteins modulating the Wnt/Frizzled signaling cascades, highly relevant for embryonic development, cell differentiation and migration, and carcinogenesis (Albrecht et al., 2021)—regulates lipid subcellular trafficking and LD biogenesis through non-canonical transcription factor effectors such as members of the TFAP2 family and the ER stress effector DDIT3/CHOP (Scott et al., 2015; Scott et al., 2018). DDIT3/CHOP is a member of the C/EBP transcription factor family; interestingly, transformation/transcription domain-associated protein (TRRAP) was identified in an independent study as a novel regulator of lipid anabolism through C/EBP alpha-mediated *de novo* synthesis of TGs in hepatocytes (Abbey et al., 2021). Other screenings have explored non-coding RNAs (Whittaker et al., 2010) and small molecule libraries (Nioi et al., 2007; Rae et al., 2011; Shin and Park, 2018) for novel regulators of LD biogenesis.

A recent genome-wide siRNA screen was performed for regulators of LD biogenesis in THP1 human macrophages (Mejhert et al., 2020). This study deployed automated extraction of 133 features from which the 21 most informative were selected, including features describing the degree of clustering of LDs, their distribution regarding their eccentricity and radial distribution, etc. The screen identified 558 hits, of which 10% were further validated in an independent small-scale screen with remarkable reproducibility, as proteins controlling LD biogenesis. The authors then conducted a proteomics analysis of THP-1-derived LD-rich fractions, looking for screen hits physically interacting with LDs; it is remarkable that a limited overlap existed between the two hit lists. Surprisingly, the authors identified the helix-loop-helix transcription factor max-like X protein (MLX) as a LD regulator (its depletion leads to large, eccentric LDs) that is found in LDs. This transcription factor partners with MLX-interacting protein (MLXIP/MondoA) and MLX-interacting protein-like (MLXIPL/ChREBP), to form glucose-sensing complexes that locate to the cytoplasm; upon glucose accumulation, the complex translocates to the nucleus to direct the expression of different glucose response genes, including potential regulators of lipid anabolism and storage (Sans et al., 2006; Stoltzman et al., 2008; Abdul-Wahed et al., 2017). MLX and MLXIP bound selectively, through C-terminal amphipathic helices in their secondary structure, to LDs rich in TAGs, but not cholesterol ester-rich LDs (Mejhert et al., 2020). Notably, MLX was found to regulate several candidates from the LD regulation screen hits, such as aquaporin 3 (AQP3), box-dependent-interacting protein 1 (BIN1) and cellular retinoic acid binding protein (CRABP2). The accumulation of LDs through oleate loading sequestered MLX and reduced its transcriptional response, while LD depletion through diacylglycerol O-acyltransferase (DGAT)1/2 RNAi increased it. Thus, this study bears particular relevance as it provides a novel model mechanism by which LD dynamics feed onto nutrient status sensing and subsequent adaptation in the cell: LD expansion and accumulation reduces the availability of a transcriptional regulator at the nucleus, dampening their positive regulation of lipid anabolism. It also highlights the versatility of LDs as a compartment buffering protein levels for different purposes (*see below*).

### A Novel Question for Functional Genomics: The Communication of LDs With Other Organelles

An emerging subfield in cell biology is the study of interorganelle communication through specialized structures, commonly referred to as membrane contact sites (MCSs), which allow for the regulated interchange of molecules and information to achieve coordinated functioning (Scorrano et al., 2019). Studying the communication between organelles beyond inferences from the functional impact of their manipulation or disruption, usually requires the integrity of cell structure, conserving spatial relationships. Biochemical purification of the specialized membrane domains of MCSs is not always possible, and most often requires extensive scales of source material (Wieckowski et al., 2009); nonetheless, proteomics approaches have provided insights onto LD-organelle communication and its molecular regulators (Krahmer et al., 2018; Freyre et al., 2019; Krahmer and Mann, 2019). Molecular proximity tools based on technologies such as fluorescence resonance energy transfer (FRET) (Periasamy, 2001) or bifluorescent complementation (BiFC) (Hu et al., 2002) reporters have been developed to explore how different MCS are established and regulated (Scorrano et al., 2019; Vallese et al., 2020). Importantly, these image-based readouts are amenable for systematic, automated screening, and have allowed for the identification not only of essential protein tethers at known MCS, but also for the detection of completely novel organelle-organelle contacts (Eisenberg-Bord et al., 2018; Shai et al., 2018).

LDs originate from specialized ER domains, and several enzymes directly regulating their growth and consumption are ER-resident proteins (Olzmann and Carvalho, 2019). Thus, it is not surprising that ER-LD physical and functional communication is pervasive and established by virtually all LDs in a cell (Cottier and Schneiter, 2022). Different, non-mutually exclusive modalities of LD-ER contacts have been conceived to accommodate the asymmetrical nature of these MCS: LD-ER contacts would consist of a conventional bilayer membrane confronting the single phospholipid layer in the LD surface, precluding other communication forms such as vesicles. “Canonical” MCSs between LDs and the ER have been inferred, whereby the surfaces of the two organelles are in close apposition, stabilized by protein tethers (Scorrano et al., 2019). Additionally, lipid “bridges” can be formed by the partial fusion of the LD surface phospholipid layer and one of the two leaflets of the ER (Schuldiner and Bohnert, 2017; Choudhary and Schneiter, 2021). This process may bear particular relevance for the regulated transfer of specific proteins from the ER to the LD (Olarte et al., 2022); substrates reaching mature LDs (i.e., “late” transfer) appear to require a specific coordination with ERES subdomains (Song et al., 20212021). A number of identified components of LD-ER contacts, including seipin/BCSL2 (Pagac et al., 2016), FIT2 (Choudhary et al., 2015), DGAT2 (Xu et al., 2012) or RAB18 (Xu et al., 2018), actively participate of LD biogenesis, supporting the notion that a major role of LD-ER contacts is the regulated synthesis and transfer of lipids from the ER to the growing LD (Herker et al., 2021). LD-ER communication might regulate not only the accumulation of specific protein subsets at LDs, but also their turnover through the process of ER-Associated Degradation (ERAD), as certain *bona fide* LD proteins are indeed specific substrates for ERAD E3 ligases (*see below*) (Ruggiano et al., 2016). Systematic protein localization screens have identified oxysterol-binding protein-related protein 5 (ORP5), an ER-resident protein, as a modulator of phospholipid composition at the LD-ER junction to regulate LD size during fatty acid accumulation (Du et al., 2020). Another hallmark example has been the screening for mutants disrupting the distribution of Prd16, a Sec14-like protein associated with a subpopulation of LDs apposed to the nucleus-vacuole junction (NVJ) in *S. cerevisiae*; complementary screens for colocalization and molecular proximity as reported by bimolecular complementation identified novel proteins (Ldo16 and Ldo45) organizing LD distribution through their cooperation with the LD-ER linker seipin (Eisenberg-Bord et al., 2018). Interestingly, LDs participate in at least two other “three way contact sites”: with the ER and vacuoles (Hariri et al., 2019), and the ER and peroxisomes (Joshi et al., 2018). All these communication structures appear to be, at least in part, related with the regulation of growth/consumption cycles of LDs. Recently, a three-way contact site has also been described in mammalian cells between LDs, early endosomes and the ER (Parton et al., 2020); its molecular features remain to be characterized.

While the close apposition of LDs to mitochondria and its link with fatty acid consumption had been early suggested (Napolitano and Fawcett, 1958), specific details about the molecular complexes regulating this apposition and their full functional significance have only recently started to be elucidated. LD subpopulations can form tight attachments with mitochondria, especially in tissues or cell types with a predominant oxidative phosphorylation metabolism (Kong et al., 2017). A key mediator of LD-mitochondria anchoring in mammalian cells is PLIN5, which also acts as a nutrient sensor through its phosphorylation by nutrient deprivation-activated kinases (Pollak et al., 2015; Benador et al., 2018). Biochemical enrichment and proteomic analyses of membranes associated with LDs recently identified the outer mitochondrial membrane MIGA2 as a tether between LDs and mitochondria, also interacting with the VAP-A/B ER protein; MIGA2-mediated tethering facilitates TG synthesis from non-lipid precursors, linking energy status in the cell to LD growth (Freyre et al., 2019). Recently, routines based on skeletonization-aided mitochondrial segmentation and statistics-assisted colocalization analysis have been developed to accurately estimate LD-mitochondria apposition from standard confocal images in mammalian cells; these workflows allow for the description of these contacts in 3D, and constitute a potential platform to explore the regulation of LD-mitochondria contacts in metazoans (Martn et al., 2022).

Peroxisomes enable β-oxidation of very long chain fatty acids in metazoans, and participate of fatty acid catabolism to its completion in yeast or plants (Alberts et al., 2014). Recent unbiased combinatorial genome-wide surveys based on BiFC discovered, among other novel LD contacts with other compartments such as the plasma membrane, a specific contact between LDs and peroxisomes; these contacts are conserved in human cells and regulated by the membrane shaper Spastin (Shai et al., 2018; Chang et al., 2019).

Contacts between LDs and subcompartments of the endosomal system have also been described (Parton et al., 2020), but their precise function remains less well understood. Previous studies provided evidence supporting a role for endosome-associated Rab proteins on LD-endosome interaction, which could contribute to LD spatial dynamics (Liu et al., 2007; Guimaraes et al., 2015). Rab proteins have also been proposed to regulate the interaction of LDs with late endosomes/lysosomes presumably to mediate their degradation (Schroeder et al., 2015; Drizyte-Miller et al., 2020).

## The LD Proteome Reveals Functions on Cell Homeostasis and Defense Beyond Lipid Metabolism

### LDs and Proteins: Who Is the Regulator? LDs as Protective Structures Against Cell Stress

Despite the conception of LDs as rather passive cell structures, evidence for the localization of proteins with enzymatic activities to the surface or close periphery of LDs was obtained several decades ago (Wigglesworth, 1966; Greenberg et al., 1991). Current models describe LD subpopulations or functional states as being largely defined by their specific proteome (Olzmann and Carvalho, 2019). Early proteomics studies—a major share of them, based on 2D-SDS-PAGE resolution and in-gel trypsinization—demonstrated that several different proteins locate to the LD surface of mammalian cells (Liu et al., 2004), *Drosophila* (Beller et al., 2006), *C. elegans* ^143^ *,* yeast (Schmidt et al., 2013) and plants (Horn et al., 2013); and that a major share of these proteins are enzymes that regulate lipid metabolism—hence, locally modulating LD formation, dynamics and consumption. These studies also identified some of the first “*bona fide*” LD proteins—meaning proteins directly and almost exclusively binding the LD surface—such as perilipins (Brasaemle et al., 1997; Londos et al., 1999; Goodman, 2009). A number of early studies also used semiquantitative proteomics to characterize changes in LDs upon different conditionals, including hepatitis C virus (HCV) infection (*see also below*); this particular study demonstrated the recruitment of RNA binding proteins participating in viral RNA replication to LDs (Sato et al., 2006).

A hallmark study applied emerging shotgun proteomics techniques to profile the proteome of LD-rich fractions from *Drosophila* larvae, yielding 51 robust hits and an additional list of 76 proteins with moderate reproducibility (Cermelli et al., 2006). Surprisingly, aside from a majority of lipid metabolism-related enzymes, membrane trafficking proteins and components of known LD-contacting organelles such as ER and mitochondria, the study found the histone paralogs H2A/B and H2Av as abundant protein components of LDs. Subsequent research demonstrated a role for LDs as safe protein buffering compartments, where extranuclear surplus of these histone paralogs can be placed at early developmental stages, avoiding a toxic accumulation outside the nucleus and making them readily available for rapid cell division (Li et al., 2012; Li et al., 2014; Johnson et al., 2018; Stephenson et al., 2021). This regulated accumulation of histones at LDs requires a LD-localized protein (Jabba), for which no clear mammalian homolog has been identified so far (Li et al., 2012). Notably, Jabba-dependent LD accumulation of histones—which have long been known to be powerful antimicrobial peptides *in vitro* (HIRSCH, 1958; Lee et al., 2009)—also confers protection from bacterial infection (Anand et al., 2012), yet another novel unexpected role for LDs (*see below*). Histones, which can be toxic for cells when mislocalized or secreted (Kawano et al., 2014; Dueva et al., 2019), have also been found on mammalian LDs (Anand et al., 2012; Bersuker et al., 2018); the mechanisms by which their recruitment is regulated in these organisms are at present unknown.

These findings seeded the concept that LDs do not only keep lipid species from accumulating at and harming other organelles, but can also constitute safe depots for proteins not directly related with lipid metabolism, and play a relevant role in preventing proteotoxicity and adapting to different stress conditions.

A first phenomenon for which a “protective” role for LDs has been suggested is ER stress, a broad term describing any imbalance between ER functional capacity and demand, which can ensue from either excessive client protein input and/or compromised ER function or integrity (Walter and Ron, 2011; Thibault et al., 2012; Volmer et al., 2013). The Unfolded Protein Response (UPR), a signaling network driven by ER stress transducers (three in higher metazoans: ATF6, PERK and IRE1) orchestrates adaptive responses that increase ER capacity, modulate different aspects of cell physiology, and ultimately promote cell apoptosis when ER stress is excessive (Hetz et al., 2020). Because ∼30% of a metazoan cell proteome is synthesized and matured at the ER (Griffing, 2010; Hetz et al., 2020), ER stress is one of the main forms of proteostasis imbalance studied to date. Further, it is a condition onto which lipid homeostasis and proteostasis converge: aberrant ER membrane composition or dysregulated lipid metabolism trigger mechanisms affecting proteostasis through the UPR, and the accumulation of misfolded proteins in the ER rewires lipid metabolism (Sriburi et al., 2004; Sriburi et al., 2007; Bommiasamy et al., 2009; Fu et al., 2011; Thibault et al., 2012; Volmer et al., 2013; Hetz et al., 2020; Ho et al., 2020). Several experimental settings inducing ER stress, including the direct pharmacological disruption of protein maturation and folding, promote the accumulation of LDs in different cell types (Fei et al., 2009; Fu et al., 2011; Lee et al., 2012). While lipid anabolism, with a predominance of phospholipid synthesis, is integral to the UPR (Sriburi et al., 2004; Sriburi et al., 2007; Bommiasamy et al., 2009; Thibault et al., 2012; Ho et al., 2020), the precise molecular mechanisms by which LD formation is induced during ER stress are not completely understood. As previously mentioned, transcription factors linked to ER stress responses such as DDIT3/CHOP (Wang et al., 1996) have been found in siRNA screens as modulators of LD biogenesis (Scott et al., 2015; Scott et al., 2018). It would also be interesting to assess the relationship of stress-induced LDs with ER subdomains organizing UPR signaling (Korennykh and Walter, 2012; Ngoc-Han et al., 2021), and whether these ER subdomains exhibit a differential lipid composition (Shen et al., 2017).

The contribution of LD formation to ER stress adaptation might be contextual: while several studies describe the upregulation of UPR markers when neutral lipid synthesis and LD biogenesis are impaired (Petschnigg et al., 2009; Chitraju et al., 2017; El Zowalaty et al., 2018), others suggest that lack of key LD formation regulators has little impact on ER homeostasis, at least under some conditions (Nguyen et al., 2017). The levels of specific LD proteins such as PLIN2 or CIDE-A are responsive to ER stress; but again, their contribution appears to be contextual, and PLIN2 depletion can have opposite effects on ER homeostasis and ER stress sensitivity in different cell models (Qiu et al., 2015; Chen et al., 2017; Morishita et al., 2021). Further, because several regulators of LD biogenesis are ER proteins, a standing question in the field is the dissection of effects due to loss of LDs, from those derived from altered ER architecture (Roberts and Olzmann, 2020).

A particular aspect of ER homeostasis surveillance that has been linked to LD dynamics, is ERAD, a turnover pathway regulating the cytoplasmic retrieval and proteasomal degradation of ER proteins ubiquitylated by specific E3 ubiquitin ligases (Carvalho et al., 2006; Vembar and Brodsky, 2008). The levels of a number of LD-localized proteins are regulated through ERAD-linked E3 ubiquitin ligases in both yeast (Dga1, Pgc1, Yeh1) and metazoans (DGAT2, c18orf32) (Ruggiano et al., 2016; Bersuker et al., 2018; Roberts and Olzmann, 2020); thus, ERAD is integrated as a mechanism to regulate the LD proteome, at least protein subsets that target LDs from the ER (Roberts and Olzmann, 2020; Song et al., 20212021; Olarte et al., 2022). Conversely, LDs might regulate ERAD contextually: ERAD regulators such as p97/VCP and its trafficking regulators UBXD8 and UBXD2 (Suzuki et al., 2012; Olzmann et al., 2013) and AUP1 (Klemm et al., 2011) are found as bona fide LD proteins by different approaches (*see below*); ERAD appears coordinated with lipid metabolism (particularly cholesterol biosynthesis) through the control of different enzymes (Foresti et al., 2013; Schumacher and DeBose-Boyd, 2021; Scott et al., 2021); and lipidostasis is required for efficient ERAD (To et al., 2017). However, clear evidence for the general requirement of LDs for ERAD remains elusive (Kong et al., 2017; To et al., 2017).

In recent years, evidence has been reported supporting that LDs contribute to proteostasis through autophagic regulation (Shpilka et al., 2015; Velázquez et al., 2016), and even through the direct management and clearance of toxic protein aggregates (Moldavski et al., 2015), at least in yeast. The exploration of context and cell type dependencies for these mechanisms in mammalian systems constitutes an important active avenue of research, particularly in cell types physiologically limited for LD formation such as neurons (Roberts and Olzmann, 2020). It is intriguing that the role of LDs as mediators of anti-proteotoxic activity described through genome-scale image-based screens by Moldavski, Schuldiner and collaborators in yeast, was associated with a sterol-related soluble metabolite pending full characterization: other soluble amphiphilic molecules such as the bile salts tauroursodeoxycholic acid and ursodeoxycholic acid have well-established chemical chaperone properties and are approved for the clinical intervention of disorders associated with loss of proteostasis (Rivard et al., 2007; Vang et al., 2014). It is currently unknown whether LD biogenesis contributes to the therapeutic activity of these widely studied molecules.

### Defining the “Core” LD Proteome

LDs are relatively easy to purify as compared to other cell compartments when using “classical” biochemical fractionation methods, due to their high buoyancy in aqueous solutions (Ding et al., 2013); these methods achieve a considerable enrichment for LDs relative to other cell compartments, in the range of ∼5,000/10,000 fold as assessed from specific markers (Zhang et al., 2012). However, current mass spectrometry methods have remarkable sensitivity and are capable of detecting >9,000 proteins in complex whole cell extracts in a single run, with a False Discovery Rate below 1% (Wang et al., 2019). This poses a challenge to distinguish “core” LD proteins—i.e., proteins specifically bound to the LD surface—, from proteins in co-purifying fragments of structures that communicate with LDs, such as ER, mitochondria, peroxisomes or endosomes. For example, certain abundant ER chaperones such as HSPA5/BiP are common contaminants appearing on LD fractions (Bersuker et al., 2018). Further, mechanical rupture during homogenization might lead to the association of proteins that can interact with the hydrophobic inner LD core, but would not normally localize to LDs in living cells (Fujimoto and Ohsaki, 2006). Bioinformatics tools can partially help to estimate the probability of a given protein to directly bind the LD phospholipid monolayer, but are still not accurate enough to identify novel *bona fide* LD proteins (Katuwawala et al., 2022).

One of the first studies aiming at specifically defining a “core” LD proteome, was reported by the Walther and Farese group (Krahmer et al., 2013). Using stable isotope metabolic labelling on *Drosophila* cells, the protein composition of the buoyant, LD-enriched fraction from a density gradient preparation was compared to all other remaining fractions as normalized to unfractionated homogenates. This analysis generates a relative enrichment profile (Protein Correlation Profile) for each protein across different cell compartments, as located by specific *bona fide* markers. These correlative approaches have been rather successful on describing the proteomes of different cell compartments including LDs, especially in studies using machine-learning approaches to describe distribution changes across conditions (Andersen et al., 2003; Itzhak et al., 2016; Mardakheh et al., 2016; Krahmer et al., 2018; Kozik et al., 2020). Indeed, an elegant refinement in the LD-PCP report, to avoid an exclusive location for each protein and better capture the distribution of proteins localizing to more than one compartment, was to generate from clustering analyses a “similarity score” for each protein profile, relative to each compartment. The study identified 111 proteins with a clear “LD-exclusive protein profile”, from a total LD-rich fraction proteome of 1,389 proteins. While the study established a first reference list of the “core” LD proteome, the strategy had limited sensitivity as only proteins reliably detected across a minimum number of fractions were considered, and could not suggest candidates for direct LD binding among proteins localizing to other compartments. Nevertheless, these strategies can be powerful to discern changes in relative LD location from and to other cell compartments when comparing different states; and importantly, they can be applied to any experimental settings and scales, as long as they are amenable for proteomics profiling. Krahmer, Mann and others combined integrative label-free proteomics and phosphoproteomics on a PCP conceptual framework, to achieve a systems-level description of how diet-induced steatosis develops in the liver, at organelle/cell compartment resolution (Humphrey et al., 2015; Krahmer et al., 2018). The study included a survey of phosphoproteome dynamics across conditions encompassing 24,523 phosphosites from 4,151 proteins at unprecedented subcellular detail; mapping to subcellular location profiles demonstrated that mitochondria and LDs are not sites highly dense for phosphoregulation. Notably, the study identified extensive reorganization of the cell proteome upon sustained exposure to high-fat diet (HFD): ∼30% of 910 proteins relocalizing at HFD were accumulating at LDs, with a remarkable overrepresentation of Golgi/secretory apparatus components, and specific subsets of organelle contact tethers. An example of the later were VPS13A and VPS13D, proteins functioning on lipid transport across MCS (Leonzino et al., 2021), whose relocation also correlated with reduced specific phosphorylation at Ser835, and Ser 2080/2,485, respectively. This correlated with a marked increase in LD-organelle contacts, such as LD-mitochondria. These studies further demonstrated how targeted proteomics approaches (for example, Parallel Reaction Monitoring (Peterson et al., 2012)) can be deployed to use these proteome changes across organelle contact structures as potential biomarkers, prognostic of disease progression (Krahmer et al., 2018; Krahmer and Mann, 2019).

A group of techniques has been developed during the last decade to identify proteins in very close proximity to, or located to the same compartment as, a certain protein marker of interest. Engineered bacterial biotin ligases can efficiently catalyze covalent bonds between biotin and lysine residues of proteins in close proximity (∼5–10 nm) *in vitro* and *in vivo*; after subsequent affinity purification, mass spectrometry allows for the identification of labelled cell proteome subsets. A variety of applications have been also derived to inform of specific events such as posttranslational modification or interorganelle communication (Roux et al., 2012; Kim et al., 2016; Cho et al., 2020; Barroso-Gomila et al., 2021; Santos-Barriopedro et al., 2021; Xiong et al., 2021). A related approach leverages on engineered ascorbate peroxidases (APEX): fusions to baits of interest generate, from biotin-tyramide and in the presence of H_2_O_2_, biotin-phenoxyl radicals that are unable to cross lipid membranes, and that rapidly form covalent adducts with tyrosine residues of proteins within a ∼20–40 nm radius (Chen et al., 2015). Because of its larger range, this technology has been preferred to characterize cell compartment-specific proteomes, as opposed to protein-specific direct interactomes (Bosch et al., 2021b; Qin et al., 2021). These methodologies have a number of advantages: they tag all proteins truly approaching the vicinity of the “bait” ligase fusion, even if such localization is transient or non-exclusive; and as the tagging proceeds *in vivo* in the cell, subsequent disruption of cell architecture has a limited impact through artifact false interactions. Further, sensitivity for detecting low abundance components is significantly increased.

Bersuker, Olzmann and collaborators fused the APEX2 ascorbate peroxidase (Lam et al., 2015) to inert variants from well-established “core” LD proteins, ATGL and PLIN2, to profile LD surface proteomes (Bersuker et al., 2018; Peterson et al., 2021). The study was rigorously designed: LD-targeted APEX2 fusions—an ATGL catalytically inactive mutant S47A, and a LD-targeting carboxyl-terminal fragment of PLIN2—were largely devoid of LD-related biological activity, background cytosolic labeling was controlled for, biotinylated LD proteomes were compared to LD-enriched total proteomes from buoyant fractions, and a stringent thresholding taking into account all these information sources was set. The study identified a high-confidence LD proteome of 153 proteins in cultured U2OS cells, 63 of which were also found in an independent hepatocyte cell model, among a buoyant fraction total proteome of 1,227 polypeptides. It is noteworthy that the LD ′core’ proteomes estimated from these two independent approaches have comparable sizes. Notable findings of the study, beyond the identification of previously uncharacterized proteins such as c18orf32 as LD proteins, include the confirmation of several Rab GTPases as LD-located proteins (as suggested by previous studies), and the identification of the ERAD pathway as a mechanism specifically regulating the LD proteome (*see previous section*).

**FIGURE 2:**
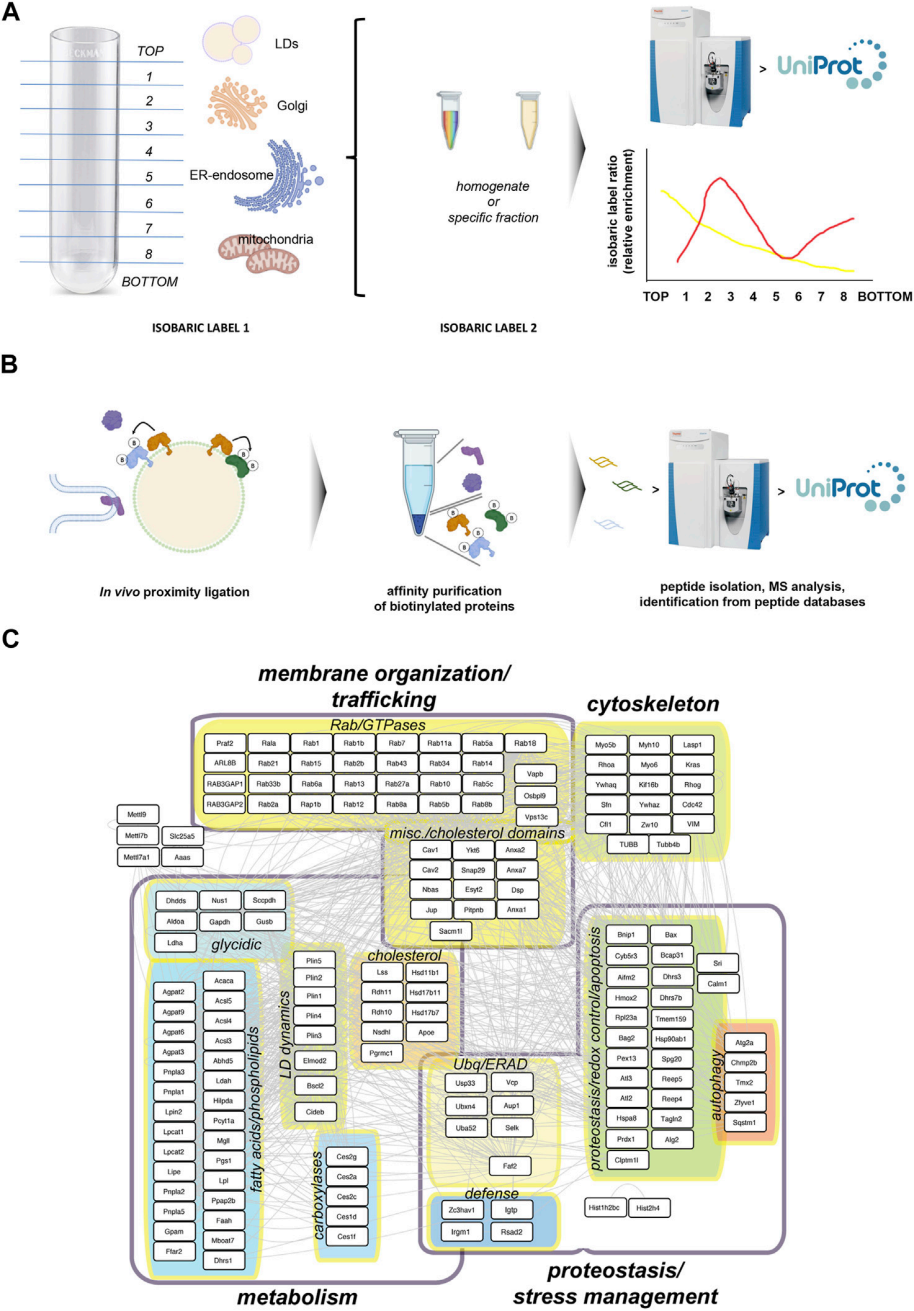
Studying the LD core proteome. **(A)** Protein Correlation Profile strategies rely on the biochemical isolation of LDs using standard biochemical fractionation from cell or tissue homogenates. An equal sample, subject to a distinct isobaric labeling from the fractionated sample, is used as reference to estimate the relative enrichment for each protein across fractions. These analyses generate relative distribution profiles, which can be related to specific cell compartments using appropriate markers, including proteins strictly localized to LDs (Krahmer et al., 2013). **(B)** Proximity ligation approaches rely on compartment-specific ligase fusion constructs (in the cartoon, orange LD protein) which catalyze the biotinylation of aminoacid residues in proteins spatially very close, while much less efficiently labeling background proteins such as cytoplasmic proteins or proteins located to a compartment appositioned to LDs. Biotinylated proteins can be affinity purified and analyzed by mass spectrometry (Bersuker et al., 2018). **(C)** A model LD ′core’ proteome combining the mouse homologs of proteins identified in the two previous studies (Krahmer et al., 2013; Bersuker et al., 2018), with descriptions of their functional grouping. Well-established defense proteins have been also included.

These studies have provided reference lists of ‘core’ LD proteins, extremely valuable to understand how responsive this subproteome is, what stimuli regulate their localization to LDs, and what molecular mechanisms underpin such localization (Figure 2). Surprisingly, one of the processes the “core” LD proteome is sensitive to, is the innate defense response against intracellular pathogens.

### LDs: Emerging Innate Immunity Effectors

Reflecting an early conception of LDs as rather “passive” lipid storage sites, a model has long been held whereby several intracellular pathogens hijack and use these organelles as convenient sources of energy and building blocks for their replication, and have even developed specific mechanisms to induce and attract LDs (van der Meer-Janssen et al., 2010; Kellermann et al., 2021). Among viral agents, a classical example is embodied by HCV: this RNA virus replicates at and is trafficked for exocytosis in close association to LDs, and its protein product HSV-NS5a induces LD biogenesis through activation of fatty acid synthase (FASN) and DGAT1—two key limiting enzymes in fatty acid *de novo* biosynthesis and triacylglycerol conjugation, respectively)—and LD tethering to replication factories through the RAB18 GTPase and accessory proteins such as Annexin A3 (ANXA3) (Herker et al., 2010; Camus et al., 2013; Dansako et al., 2014; Loizides-Mangold et al., 2014). Other Flaviviridae such as Dengue viruses are also prominent examples of viruses specifically interacting with host LDs during infection (Randall, 2018; Zhang et al., 2018). Intracellular bacterial Gram^−^ and Gram^+^ pathogens, such as *Chlamydia trachomatis* and *Mycobacterium tuberculosis* (*Mtb*), induce LD formation and engage in complex interactions through their surrounding phagophore with these organelles, presumably to utilize their resources (Cocchiaro et al., 2008; Peyron et al., 2008; Daniel et al., 2011; Armstrong et al., 2016; Daniel et al., 2016; Recuero-Checa et al., 2016; Mulye et al., 2018; Sharma et al., 2018; Roque et al., 2020). Eukaryotic intracellular parasites (Plasmodiidae*,* Trypanosomatidae*,* Sarcocystidae) also induce the formation of LDs in the host cell, can liberate lipases to obtain free lipids from host LDs, and actively uptake these free lipids for the completion of their biological cycle (Rodríguez-Acosta et al., 1998; Nolan et al., 2017; Vallochi et al., 2018; Bosch et al., 2021a).

However, this model of LDs as solely “comforting pantries” for intracellular invaders does not fit with different lines of evidence. First, formation of LDs is actively promoted in the cell by the activation of pathogen pattern recognition receptors (PRRs) by their microbial invader product (pathogen-associated molecular pattern, PAMP), such as bacterial surface lipopolysaccharides (LPS) and glycolipids (Nicolaou et al., 2012; D’Avila et al., 2006) or nucleic acids (Monson et al., 2021), through both canonical innate immunity signaling pathways like type II interferon, as well as other stress response axis such as Hypoxia Inducible Factors (Knight et al., 2018). Indeed, killed pathogens can strongly induce LD formation (Rabhi et al., 2016). Control of lipid metabolism and the competency for LD biogenesis are important for cells to counteract the invasion by pathogens (Mulye et al., 2018; Sharma et al., 2018). Specific defense proteins (viperin/RSAD2 or IGTP) and lipid intermediates (arachidonic acid), which take part on antimicrobial responses and immunity regulation, have been identified as localizing to LDs (Bougnères et al., 2009; Hinson and Cresswell, 2009; Dichlberger et al., 2014). Importantly, purified LD-rich fractions bear a strong, largely protein-mediated, conserved antimicrobial activity, and this activity is significantly enhanced by LPS sensitization (Anand et al., 2012). It does make sense that evolution would favor the wiring of pathogen sensing and defense systems to such a preferred subcellular location as LDs (Bosch et al., 2021a).

Using an experimental system where LD formation is induced in liver upon starvation and LPS administration in mice (thus minimizing effects from reduced food intake upon exposure to LPS), we applied multiplexed isobaric labeling and tandem mass spectrometry to profile the proteome of purified hepatic LDs, and its response to PRR activation (Bosch et al., 2020a). We observed a significant remodeling of the LD proteome in response to LPS: across 3,392 robustly identified proteins, we observed significant changes in the relative levels of 689 proteins (317 enriched, 312 reduced), which exhibited a limited overlap with those changes observed in unfractionated liver homogenates, suggesting rather specific changes. These included an estimated ∼30% of a reference “core” LD proteome, built from the studies summarized in the previous section (Krahmer et al., 2013; Bersuker et al., 2018), whereby all 5 mouse perilipins, 19 Rab GTPases, key regulators of phospholipid and fatty acid metabolism, and central regulators of ERAD-mediated protein turnover such as AUP1 and VCP/p97, were responsive to LPS. Functional annotation enrichment evidenced profound changes in the communication of LDs with other organelles: a clear disconnection from mitochondria was occurring, in accordance with the reduced levels observed for PLIN5, a key LD-mitochondria tether (Kien et al., 2022); in contrast, data suggested a potential increased interaction with the endosomal/multivesicular body compartment (Drizyte-Miller et al., 2020) upon LPS administration.

Leveraging on variability across biological replicates, which can capture relevant biochemical relationships (Meunier et al., 2007), hierarchical clustering yielded functionally coherent protein groups. These included a robustly upregulated cluster of innate immunity proteins—some of them, already demonstrated to localize to LDs, such as viperin/RSAD2 and IGTP (Bougnères et al., 2009; Hinson and Cresswell, 2009)—together with known “core” LD proteins PLIN2 (Jiang and Serrero, 1992) and Rab18 (Xu et al., 2018). Of note, PLIN5 overexpression in LPS-treated cells reduced the antimicrobial response associated with LD induction; a mutually excluding spatial localization pattern at LDs has been previously reported for PLIN2 and PLIN5, which could be outcrowding upon overexpression any PLIN2-associated complexes (Gemmink et al., 2018). The cluster also included interferon-responsive GTPases (IIGP, TGTP1, IFI47) of as yet unknown specific functions, which were subsequently proven to localize to LDs. Immune-related GTPases remain an incompletely characterized component of the innate defense system in cells against extracellular pathogens (Rafeld et al., 2021).

An additional member of the PLIN2-nucleated cluster was the E3 ubiquitin ligase mysterin/RNF213, which contributes to the regulation of LD dynamics (Sugihara et al., 2019). This enzyme has been recently reported as the first E3 ligase capable of ubiquitylating bacterial LPS, presumably to target bacteria and their products for lysosomal degradation (Otten et al., 2021). Further, RNF213 assembles in oligomers on the surface of LDs upon interferon-induced conjugation with the small protein modifier insulin-stimulated gene 15 (ISG15). LD-bound RNF213 complexes act themselves as sensors of ISGylated proteins, and display defense activity against different intracellular pathogens, including *Listeria monocytogenes* or herpes simplex virus type 1 (Thery et al., 2021).

Cathelicidin antimicrobial peptide (CAMP, LL-37) is an amphiphilic peptide with prominent roles in innate defense against bacterial infection (Bals et al., 1998; Vandamme et al., 2012). Its precursor protein CAP-18 was also found to be a member of the LPS-responsive PLIN2 cluster (Bosch et al., 2020a). Extensive validation assays confirmed that CAMP accumulates at LDs and coimmunoprecipitates with PLIN2, that CAMP expression is required for LDs to confer antimicrobial protection to cultured cells, and that artificial restriction of CAMP localization to LDs by fusion to a heterologous LD-targeting sequence preserved its intracellular antimicrobial activity (Bosch et al., 2020a). Notably, the LD binding domain of CAMP appears to be embedded in its N-terminal signal peptide; constructs lacking these sequence stretches do not accumulate at LDs, nor do they enter an efficient secretory route, suggesting that its accumulation at LDs is integral to its natural biosynthesis and release.

While exhibiting a distinct clustering pattern, histones were also found as proteins relatively enriched on LDs upon exposure to LPS. As mentioned above, histones have an intrinsic antibacterial activity (Miller et al., 1942) that synergizes with CAMP (Doolin et al., 2020), and play an important role in *Drosophila* antimicrobial defense upon recruitment by the Jabba protein (Anand et al., 2012); they are an important component of neutrophil extracellular traps (NETs) both regarding defensive activity as well as tissue toxicity (Volker et al., 2004). How LD accumulation of histones is regulated in mammalian cells, for which a Jabba homolog has not been identified, and how that mechanism is wired onto innate immune signaling, is currently unknown.

Thus, LDs emerge as strategic sites to safely but effectively mount a first-line integrated defense against intracellular invading microorganisms; indeed, in this study, a direct visualization of the engagement of LDs with intracellular bacteria, with evidence of disruption of bacterial membrane continuity, was provided (Bosch et al., 2020a). Simultaneously, LDs respond to defense activation signals by disconnecting from mitochondria, both contributing to metabolic rewiring of the cell to confront infection challenge, and protecting mitochondria from damage.

**FIGURE 3:**
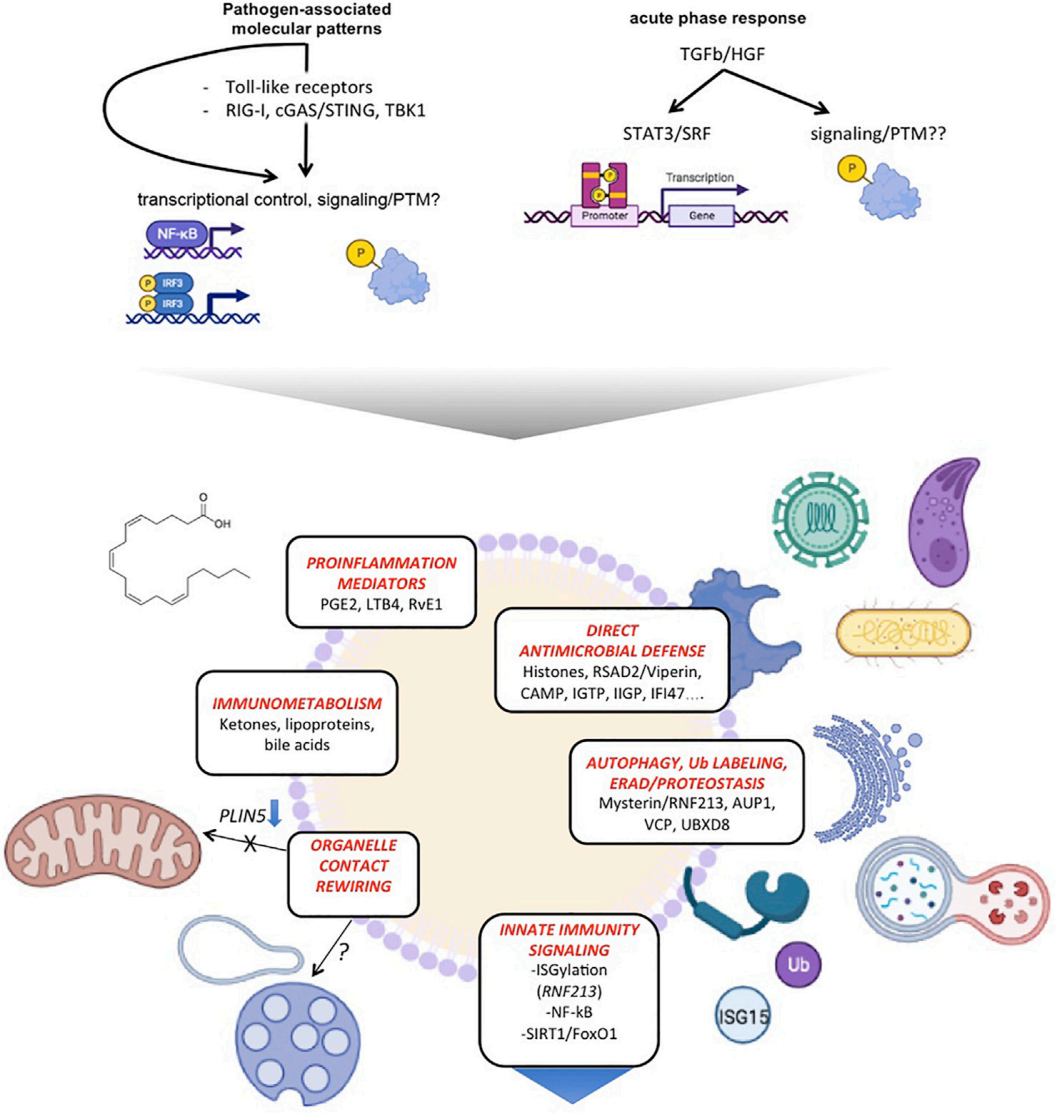
LDs emerge as hubs coordinating antimicrobial defense, innate immune signaling and metabolic rewiring. The scheme summarizes distinct functions of LDs on innate immunity and their molecular players.

These findings were validated in different cell types, including macrophages. Subsequent studies using a sepsis model (cecal ligation and puncture) support these are general principles (de Andrade et al., 2014; Bosch et al., 2020a) (Figure 3). However, distinct features may exist when considering specific cell type models, infecting microorganisms or experimental settings. Recent quantitative proteomics studies described the differences induced in proteins co-purifying with LDs in macrophages infected with live *Mtb* bacteria, and compared them to those identified from macrophages exposed to killed bacteria (Menon et al., 2019). The overlap of these datasets with those described previously is limited, in part due to their lower relative coverage; it is likely that specific pathogens from different clades also promote specific LD reprogramming. Interestingly, the study found, as specifically associated with active infection, functional categories such as vesicular trafficking and lipid metabolism, reflecting the active hijacking of these host functions by live invading bacteria. An example validated by the authors is the ADP Ribosylation Factor-Like GTPase 8B (ARL8B), a small GTPase that participates of endolysosomal trafficking (Khatter et al., 2015) and contributes to the virulence of virulent *Mtb* types (Michelet et al., 2018).

While limited on its coverage (a total 107 proteins were identified with high confidence, using label-free analyses), a report on the proteomic composition of LD-rich fractions purified from *C. trachomatis*-infected epithelial cells found changes associated with LD metabolic reprogramming (both increased lipid usage and anabolism), and identified PLIN2 as a perilipin protein specifically enriched upon infection (Saka et al., 2015). Of note, the Cap1, CTL0882, and IncG proteins from the inclusion membrane of the invading microorganism were also found associated with LDs, suggesting a role for them in modulating the interaction with these organelles. Another recent report described the association of *Mtb* proteins with LDs through amphipathic helices (Armstrong et al., 2018); studies describing the interaction of proteins from the invading microorganism with host LD proteomes will help to better understand these complex interactions.

## Future Directions

### Realizing the Promise of High Content Screening to Understand LD Biology Across Scales

While cell-based high-content screenings on LD biology have been fruitful, recent developments of these technologies could allow for the systematic interrogation of how these structures are regulated in contexts closer to physiological conditions, and how they relate to many other tissue components and markers. Automated culture and high-content analyses of organoids that capture tissue microenvironment properties have been reported (Lukonin et al., 2020; Lukonin et al., 2021). Recent technologies allow for the multiplexing of several markers at single-cell resolution (Gut et al., 2018), even within tissue samples preserving native architecture (Rodríguez-Pena et al., 2020; de Andrea et al., 2021; Jiménez-Sánchez et al., 2022). These advancements could provide novel insights onto non-cell autonomous principles, population and microenvironment-dependent mechanisms, and phenotypic states, associated with LD regulation. It must be considered that high-throughput, automated analysis-based techniques for electron microscopy imaging are also coming of age (Bykov et al., 2019; Heinrich et al., 2021; Xu et al., 2021); it is likely that these technologies will enable us to learn novel central principles on the function and regulation of LDs.

### LD Lipidomics: A Lot to Find Ahead?

An open avenue of research that has perhaps been relegated by the predominant focus on studying LD-associated proteomes, is the detailed profiling of the different lipid species composing either the phospholipid monolayer or the neutral core of LDs. This “two-part” ultrastructure depiction is deceivingly simple, and the precise nature of the lipid species in either LD subcompartment can have a robust effect on the biological role of these organelles. For example, we do know that in general terms, phosphatidylcholine to phosphatidylethanolamine species ratio and local *de novo* phospholipid synthesis are important parameters determining the size and coalescence of LDs (Fei et al., 2011; Krahmer et al., 2011). Phosphatidylinositol-4 phospate (PI_4_P) species accumulate in large LDs of cells depleted of the ORP5 regulator, which has been proposed to supply phosphatidylserine from PI_4_P (Du et al., 2020). However, lipidomics analyses suggest that >150 different phospholipid species can be detected in LD-rich fractions, apart from neutral lipids (Bartz et al., 2007); the LD phospholipid monolayer appears enriched with phosphatidylcholine and phosphatidylethanolamine species and depleted of sphingomyelin and phosphatidylserine as compared to other cell membranes. We are far from having a complete picture as to how all these different phospholipid species behave during responses of LDs to different conditions, and how their relative amounts, organization and packaging influence the dynamics of the LD proteome (Prévost et al., 2018; Čopič et al., 2018; Olzmann and Carvalho, 2019). It must also be noted that an analogous problem to that faced by proteomics studies arises regarding the potential co-purification of membrane fragments from other compartments, which may be in turn differential when comparing different functional states.

Similarly, the composition of the neutral core can vary substantially depending on the predominating substrate used to build LDs, especially in particular cell types such as macrophages: the LD can be formed predominantly from cholesterol esters or triacylglycerides; this can have a profound impact on protein association, usage dynamics and even ultrastructural organization (Fujimoto and Parton, 2011; Mejhert et al., 2020). The neutral core of LDs can also contain esters of other lipid species such as the ether monoalk (en)yl diacylglycerol (Bartz et al., 2007) or retinol esters (Molenaar et al., 2021); while their biogenesis is regulated by distinct mechanisms, we have a limited understanding of their functional particularities.

Finally, lipid species with specific functional roles, such as signaling modulation, metabolic homeostasis or proteostasis, or innate immunity are synthesized at LDs (Dichlberger et al., 2014; Moldavski et al., 2015). While thousands of distinct features can be identified in a standard mass spectrometry run from cell samples, current identification ranges lie on the hundreds (Kind et al., 2013). Tools integrating genome information (Linke et al., 2020) could help in enabling the systematic identification of novel, functionally relevant species associated with LDs.

### Next Steps in LD Proteomics: Systematic Profiling of Posttranslational Modifications

LD proteomes respond to different conditions, but how input information is conveyed is still not fully understood. Posttranslational modifications (PTMs) constitute a versatile means to modify the function, localization, stability, and interactions of proteome subsets in a coordinated manner. Protein phosphorylation comprises a large share of PTMs, driving signaling pathways for the transduction of different cues, including several modulating LD functions. An example is the regulation of LD growth and dynamics by different nodes of nutrient state sensing networks in the cell (Bosch et al., 2020b). Fasting promotes protein kinase A-dependent phosphorylation of PLIN1 and PLIN5, among other LD-located substrates, releasing the activity of lipases such as ATGL and HSL (whose activity is also promoted by PKA-dependent phosphorylation) (Egan et al., 1992; Brasaemle et al., 2000; Tansey et al., 2003; Pagnon et al., 2012; Pollak et al., 2015). 5′ AMP-activated protein kinase (AMPK), another major nutrient sensing kinase triggering starvation stress responses such as autophagy (Kim et al., 2011) and suppression of lipid storage (Brusq et al., 2006), can also stimulate LD lipolysis (Ahmadian et al., 2011). Regulatory kinases may locate directly on the LD surface: the serine/threonine kinase 25 (STK25) is a stress-related kinase which promotes hepatocyte lipid accumulation (Amrutkar et al., 2016); profiling of the STK25-related phosphoproteome co-fractionating with LDs revealed 130 proteins and 60 target phosphosites, enriched for regulators of peroxisomal function and proteostasis responses, providing a mechanistic basis for the involvement of this kinase in non-alcoholic fatty liver disease progression (Nerstedt et al., 2020). Despite these significant advances, we have a limited understanding regarding the complete architecture of these signaling networks, and how they interplay with each other (Djouder et al., 2010; Bosch et al., 2020b). Reflecting the increasing complexity found in combinatorial PTM-driven signaling and proteome regulation (Aggarwal et al., 2021), recent studies confirm that LD proteins are substrates for, and/or regulators of, acetylation (Qian et al., 2017; Nguyen et al., 2019), palmitoylation (Suzuki et al., 2015), ubiquitylation (Zhang et al., 2018; BasuRay et al., 2019; Sugihara et al., 2019), ISGylation (Thery et al., 2021), or UFMylation (Eck et al., 2020). Proteomics procedures hold a key to globally understand the dynamics and function of these PTMs, but a majority of strategies have relied on the affinity purification of peptides bearing a specific PTM before its analysis by mass spectrometry (Murray et al., 2019; Nelson et al., 2021; Paulo and Schweppe, 2021; Rivera et al., 2021; Hsieh et al., 2022). These procedures have enabled the charting of signaling networks and their dynamics with unprecedented resolution, but they are limited to describe the relationships and interplay of different PTMs among each other, and often require substantial sample amounts. Novel computational frameworks leverage on the capacity of current shotgun proteomics, for which typically a major share of raw spectra cannot be readily assigned to a specific peptide or protein record (Skinner and Kelleher, 2015; Griss et al., 2016). Methods iteratively interrogating deviations from expected molecular masses for a given peptide can chart virtually all possible PTMs that could be detected in conventional closed searches (Kong et al., 2017; Bagwan et al., 2018). While the application of these strategies on quantitative studies based on isobaric labeling might pose additional changes, in principle they could enable the profiling of all PTMs modulating the LD proteome across different conditions. The integration of these datasets with orthogonal information from functional profiling studies (especially regarding those enzymes regulating PTMs) could provide novel insights onto the principles regulating LD biological roles.

The advancements brought by functional genomics and molecular profiling to all fields in cell biology are particularly prominent in LD research, as their characterization may have lagged as compared to other cell compartments, and some of their emerging functions (proteostasis, cell autonomous defense) were particularly unexpected. Novel unbiased technologies will surely contribute to uncover new systems-level aspects of the roles of LDs in cell function and their emerging relationships with other cell structures, such as the nucleus and the secretory apparatus.
